# Relevance and regulation of alternative splicing in plant secondary metabolism: current understanding and future directions

**DOI:** 10.1093/hr/uhae173

**Published:** 2024-07-02

**Authors:** Zihan Xu, Ying Xiao, Jinlin Guo, Zongyou Lv, Wansheng Chen

**Affiliations:** Research and Development Center of Chinese Medicine Resources and Biotechnology, Shanghai University of Traditional Chinese Medicine, Shanghai 201203, China; Research and Development Center of Chinese Medicine Resources and Biotechnology, Shanghai University of Traditional Chinese Medicine, Shanghai 201203, China; College of Medical Technology, Chengdu University of Traditional Chinese Medicine, Chengdu 611103, China; Chongqing Key Laboratory of Sichuan-Chongqing Co-construction for Diagnosis and Treatment of Infectious Diseases Integrated Traditional Chinese and Western Medicine, Chengdu University of Traditional Chinese Medicine, Chengdu 611130, China; Key Laboratory of Characteristic Chinese Medicine Resources in Southwest China, College of Pharmacy, Chengdu University of Traditional Chinese Medicine, Chengdu 611103, China; Research and Development Center of Chinese Medicine Resources and Biotechnology, Shanghai University of Traditional Chinese Medicine, Shanghai 201203, China; Research and Development Center of Chinese Medicine Resources and Biotechnology, Shanghai University of Traditional Chinese Medicine, Shanghai 201203, China; Department of Pharmacy, Changzheng Hospital, Second Military Medical University, Shanghai 200003, China

## Abstract

The secondary metabolism of plants is an essential life process enabling organisms to navigate various stages of plant development and cope with ever-changing environmental stresses. Secondary metabolites, abundantly found in nature, possess significant medicinal value. Among the regulatory mechanisms governing these metabolic processes, alternative splicing stands out as a widely observed post-transcriptional mechanism present in multicellular organisms. It facilitates the generation of multiple mRNA transcripts from a single gene by selecting different splicing sites. Selective splicing events in plants are widely induced by various signals, including external environmental stress and hormone signals. These events ultimately regulate the secondary metabolic processes and the accumulation of essential secondary metabolites in plants by influencing the synthesis of primary metabolites, hormone metabolism, biomass accumulation, and capillary density. Simultaneously, alternative splicing plays a crucial role in enhancing protein diversity and the abundance of the transcriptome. This paper provides a summary of the factors inducing alternative splicing events in plants and systematically describes the progress in regulating alternative splicing with respect to different secondary metabolites, including terpenoid, phenolic compounds, and nitrogen-containing compounds. Such elucidation offers critical foundational insights for understanding the role of alternative splicing in regulating plant metabolism and presents novel avenues and perspectives for bioengineering.

## Introduction

**Figure 1:**
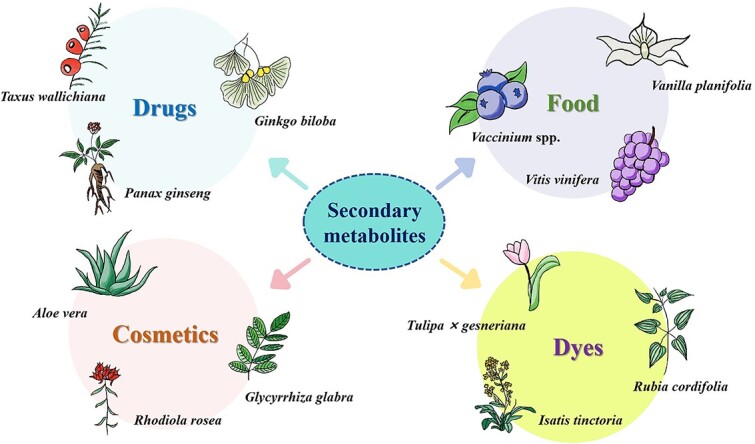
Secondary metabolites from plants play a crucial role in various aspects of daily life and find wide applications in medicine, chemistry, food, and cosmetics. For instance, secondary metabolites from medicinal plants like *Panax ginseng*, *Ginkgo biloba*, and *Taxus wallichiana* serve as key ingredients in pharmaceutical formulations, offering therapeutic benefits for treating a range of diseases. Additionally, plants such as *Isatis tinctoria*, *Rubia cordifolia*, and *Tulipa* × *gesneriana* are utilized as natural dyes to impart vibrant colors to textiles and other materials. In the food industry, plant secondary metabolites are incorporated into products for flavoring, preservation, and nutritional enhancement, with examples including *Vanilla planifolia*, *Vitis vinifera*, and various *Vaccinium* species. Furthermore, these compounds are integral to the cosmetics industry, where their beneficial properties and pleasant aromas are utilized in skincare, perfumes, and other beauty products. Common plants like *Rhodiola rosea*, *Glycyrrhiza glabra*, and *Aloe vera* contribute to this aspect.

In nature, plants do not exist in isolation but rather interact with other organisms and their surrounding environment, inevitably influenced by it. When faced with environmental stress, plants produce secondary metabolites to combat instability and sustain their normal growth and development process. These secondary metabolites are highly valuable to humans, finding applications across various industries, including the chemical, cosmetic, pharmaceutical, and spice sectors [1–4] (Fig. 1). For instance, artemisinin, derived from *Artemisia annua*, is a sesquiterpene compound renowned for its significant medical benefits, including antimalarial properties [5], as well as its potential in treating tuberculosis [6] and diabetes [7]. Similarly, vinblastine and vincristine, derived from *Catharanthus roseus*, exhibit potent anticancer effects [1], thus playing a pivotal role in the pharmaceutical industry. Furthermore, linalool, a common essential oil component, finds widespread use in the cosmetics industry [2].

**Figure 2:**
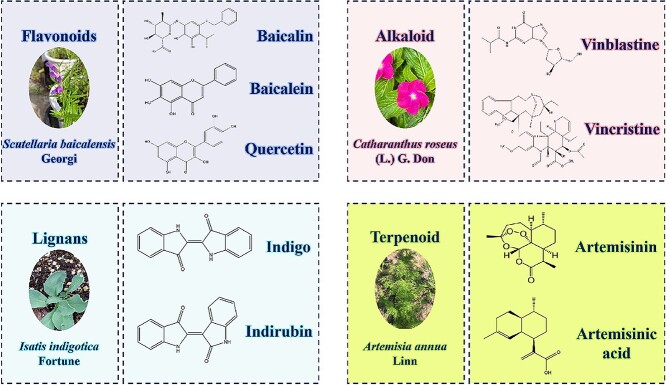
Secondary metabolites of common Chinese medicine and their types. Terpenoids from *Artemisia annua*: artemisinin and artemisinic acid; alkaloids from *Catharanthus roseus*: vinblastine and vincristine; lignans from *Isatis indigotica* Fortune: indigo and indirubin; flavonoids from *Scutellaria baicalensis* Georgi: baicalin, baicalein and quercetin.

Secondary metabolism refers to the process by which organisms synthesize non-essential substances for immediate survival and store secondary metabolites. These secondary metabolites are derived from primary metabolites, serving as precursors to synthesize compounds that lack a direct physiological function but contribute to various biological activities. This process is known as secondary metabolism, and the resulting products are termed secondary metabolites. Secondary metabolites possess protective properties that aid plants in surviving and adapting to their environment [8]. Secondary metabolites are characterized by a complex molecular structure, unique metabolic pathway, involvement of numerous enzymes, typically low yields, and often unclear physiological functions [9]. The chemical structures of secondary metabolites are complex, as are their synthesis pathways. Secondary metabolite biosynthesis pathways can be categorized into several extensions of primary metabolic pathways, including glucose metabolism, the shikimic acid pathway, amino acid pathways, and acetic acid pathways. Plant hormones play a crucial role in influencing the accumulation of secondary metabolites. Due to the diversity of secondary substances, their metabolic reactions vary significantly, often occurring only in specific species, organs, or tissues under particular environmental and temporal conditions. Various primary metabolic pathways, such as sugar metabolism, the tricarboxylic acid (TCA) cycle, lipid metabolism, amino acid metabolism, and terpene and steroid metabolism, serve as the foundation for secondary metabolic pathways [4]. These pathways are broadly classified into three groups based on differences in chemical structure and biosynthesis: terpenoids, phenolic compounds, and nitrogen-containing compounds [10] (Fig. 2).

This adaptation in plants is regulated at various molecular levels, including post-transcriptional regulation, such as alternative splicing. Further research into the mechanism of alternative splicing will aid in enhancing the expression and accumulation of beneficial chemical components, improving plant growth, yield and biomass production, and enhancing plant stress resistance. Alternative splicing is a prevalent post-transcriptional regulatory mechanism in multicellular organisms, crucial for regulating gene expression and protein synthesis. It contributes to the diversification of proteins in eukaryotic organisms and significantly expands the coding capacity of the genome [11]. In this paper we summarize and provide an updated understanding of the effects of alternative splicing on secondary metabolites. We demonstrate how alternative splicing regulates physiological and biochemical processes, such as gene function and metabolism (Table 1).

**Table 1:** Genes affecting plant metabolism and related physiological processes by alternative splicing.

Factors related to alternative splicing		Spliced isoform	Function	Reference
Secondary metabolism	Terpenoid	CsLIS/NES-1, CsLIS/NES-2	CsLIS/NES-1 is highly expressed in chloroplasts and ultimately mediates linalool biosynthesis. CsLIS/NES-2 is expressed in the cytoplasm and is involved in biosynthesis of nerolide.	[49]
		OsWRKY62.1, OsWRKY62.2; OsWRKY76.1, OsWRKY76.1	OsWRKY62.1 and OsWRKY76.1 inhibit the biosynthesis of diterpene compounds momilactone A, phytocassane B, and phytocassane C in *Oryza sativa*. OsWRKY62.2 and OsWRKY76.2 can reduce repressor activity in biosynthesis of diterpene	[50]
		IrKSL3, IrKSL3a	IrKSL3a can produce isopimaradiene and miltiradiene. IrKSL3 only produces miltiradiene.	[51]
	Phenolic compounds	VvMYBA1, VvMYBA1-L	VvMYBA1 promotes accumulation of anthocyanin. VvMYBA1-L inhibits the flesh coloration of grape and petal coloration.	[62]
		VvMYBA6, VvMYBA6.1	VvMYBA6 can activate biosynthesis of anthocyanin. VvMYBA6.1 cannot activate biosynthesis of anthocyanin.	[63]
		IbMYB1a, IbMYB1b	IbMYB1a induces anthocyanin production. IbMYB1b does not have this function, but can increase mRNA expression levels.	[64–66]
		cBrMYB2, gBrMYB2	gBrMYB2 with short introns promotes anthocyanin synthesis resulting in a purple phenotype. cBrMYB2 with long intron 1 produces normal color in *Arabidopsis*.	[67, 68]
		Full-length CmbHLH2, truncated CmbHLH2	Full-length CmbHLH2 can activate anthocyanin biosynthesis genes and induce pigment accumulation. Truncated CmbHLH2 is located in the cytoplasm and cannot interact with CmMYB6.	[71]
	Nitrogen-containing compounds	Full-length PR3b, truncated PR3b	Full-length PR3b has high chitinase activity. The enzyme-specific activity of truncated PR3b was significantly reduced.	[74]
		Full-length SGD, truncated SGD	Full-length SGD has glucosidase activity. Truncated SGD has a truncated C-terminal and lacks glucosidase activity.	[75]
Phytohormones	JA	α-Type LOX, β-type LOX	The α-type and β-type CsLOXs have different expression patterns and may compete or compensate in regulation of each other, thus affecting the content of JA and altering the content of secondary metabolites.	[78]
		JAZ10.1, JAZ10.3, JAZ10.4	Full-length JAZ10.1, partially truncated JAZ10.3 and JAZ10.4 with complete deletion of the Jas domain. JAZ10.4 showed JA insensitivity phenotype, male root sterility and root anti-JA elongation.	[80–82]
		JAZ1.1, JAZ1.2, JAZ1.3	Under induction of JA, CsJAZ1-3 interacted with CsJAZ1-1 and CsJAZ1-2 to repress gene expression involved in biosynthesis of flavan-3-ol.	[83]
		JAZ13a, JAZ13b, JAZ13c	Overexpression of OsJAZ13a resulted in attenuation of root by methyl JA.	[84]
	ABA	ABI3-α, ABI3-β	The ABI3-α transcript encodes a full-length ABI3 protein, and the ABI3-β transcript encodes a truncated protein that contains two of the four functional domains. ABI3-β transcript accumulates at the end of seed maturation.	[88, 89]
Stress		IDD14α, IDD14β	IDD14α can bind to downstream *QQS* genes to promote starch accumulation. IDD14β, which lacks a DNA-binding domain, does not have this function.	[100, 101]
		RCD1.1, RCD1.2	The correct shear body of RCD1.1 is helpful to improve the salt tolerance of plants under salt stress and maintain normal physiological activities.	[102]
Biomass		ScMYAS1-2, ScMYBAS1-3, ScMYBAS1-4 and ScMYBAS1-5	Overexpression of ScMYBAS1-2 and ScMYBAS1-3 spliced transcripts in rice promoted changes in plant growth in rice and drought conditions.	[105]

(*Continued*)

**Table 1:** Continued

Factors related to alternative splicing		Spliced isoform	Function	Reference
Primary metabolism		MaMYB16L, MaMYB16S	MaMYB16L can bind the promoter of the starch degradation gene, inhibit starch degradation and delay banana fruit ripening. MaMYB16S lacks the DNA-binding domain and cannot bind these promoters, antagonizing MaMYB16L.	[109]
		LeCBDGK, LeDGK1	LeCBDGK has a calmodulin binding domain that binds to calmodulin and is activated by calcium ions. LeDGK1 does not have a calmodulin binding domain and cannot bind to calmodulin.	[112]
Trichome		AlNAP1-AS1, AlNAP1-AS2	Truncated AlNAP1-AS1 and AlNAP1-AS2 lack multiple exons and cannot rescue the *nap1* mutant in *Arabidopsis* compared with full-length types.	[115]
		CPL4-α, CPL4-β	Both CPL4-α and CPL4-β contain conserved amino acid markers that are required to interact with bHLH transcription factors.	[122]
		SmD3-a and SmD3-b	SmD3-b may be a major component of the spliceosomal snRNP in *Arabidopsis*, but the function of SmD3-a may be redundant.	[123]

## Pre-mRNA splicing and roles of alternative splicing in plants

### Mechanism and types of mRNA precursor splicing

Transcription and translation constitute the entire process of gene expression, with RNA splicing being a particularly crucial step in this process. Pre-mRNA splicing serves as a central mechanism for regulating gene expression in eukaryotes [12]. The mRNA splicing process is a post-transcriptional processing mechanism wherein introns within pre-mRNA are excised at splicing sites, and exons are sequentially joined to form mature mRNA. This process involves assembly, rearrangement, and catalytic activity. Five small nuclear ribonucleoproteins (snRNPs), rich in uracil, collectively known as the spliceosome, facilitate mRNA splicing. These spliceosome subtypes include U1, U2, U4, U5, and U6. The snRNPs are responsible for removing the introns from pre-mRNA to mature mRNA. The RNA within pre-mRNA, termed small nuclear RNA (snRNA), is a vital component of the snRNP complex, typically around 150 bases in length. Additionally, various Sm proteins or Sm-like proteins, which bind to conserved Sm sequences, are crucial constituents of snRNP. Sm proteins belong to a highly conserved family of RNA-binding proteins (RBPs). In eukaryotes, Sm proteins associate with RNAs to form snRNPs, playing a pivotal role in gene regulation.

**Figure 3:**
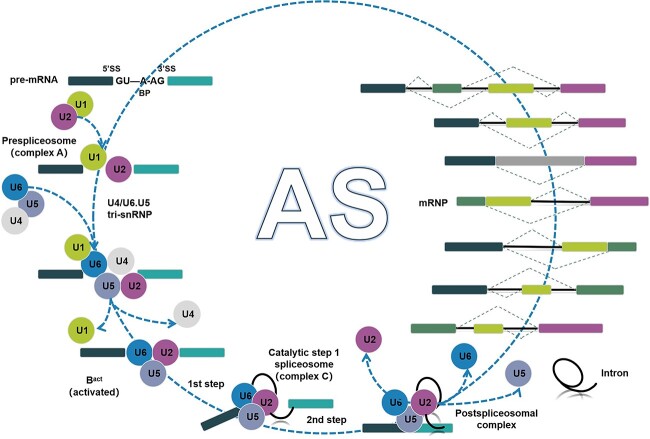
The mechanisms of splicing and alternative splicing involve the coordinated action of U1, U2, U4, U5, and U6 spliceosomal proteins. Initially, U1 recognizes the 5′ splice site of the intron, while U2 recognizes the 3′ splice site, forming complex A. Subsequently, U6 displaces U1, and U5 along with U2 positions itself at the 5′ splice site and the branch site, forming the spliceosome. Upon the dissociation of U1–U4, U2–U6 forms a catalytic center. At this stage, U5 recognizes the two exon splice sites, leading to two transesterification reactions catalyzed by U2–U6, thereby ligating the exons and completing the splicing process. Alternative splicing gives rise to various types of splice variants, depicted in seven schematic representations. In these schematics, the gray box represents a constitutive exon, the colored thick square signifies an alternatively spliced exon, the solid black line denotes the exon, the gray box represents an exonized intron, and the dashed upper and lower lines illustrate the alternative processing of exons.

The proper assembly of snRNPs is a crucial prerequisite for the formation of RNA splicing complexes. While U6 snRNA is transcribed by RNA polymerase III, other snRNAs are transcribed by RNA polymerase II. U6 snRNA combines with other proteins in the nucleus to form U6 snRNP, whereas other snRNAs are initially transported to the cytoplasm. There, they associate with other proteins to form snRNPs before entering the nucleus as subunits. This process ensures the completion of RNA splicing within the nucleus [13]. RNA splicing is a tightly regulated process, involving not only splicing sites and spliceosomes but also numerous regulatory elements known as splicing regulatory elements (SREs). Pre-mRNA contains multiple exons and introns, and the selection of splicing sites is collectively regulated by *cis*-splicing regulatory elements and *trans*-splicing regulatory factors. *Cis*-acting elements, based on their relative position and activity, can be categorized into exon splicing enhancers (ESEs), intron splicing enhancers (ISEs), exon splicing silencers (ESSs), or intron splicing silencers (ISSs). These *cis*-acting elements recruit splicing factors to either facilitate or inhibit the recognition of adjacent splicing sites [14] (Fig. 3).

There are two distinct types of splicing of mRNA precursors (Fig. 3). The first is constitutive splicing, which represents the fundamental mechanism of RNA splicing. The spliceosome accurately recognizes the splicing site, excises the intron entirely from the mRNA precursor, and subsequently joins the exons to form mature mRNA in a standardized manner. In this scenario, splicing alterations are limited, and each transcription unit typically yields a single mature mRNA [14]. The second type is alternative splicing, where the same pre-mRNA can undergo splicing by selecting different splice sites, resulting in the formation of various mature mRNAs and thereby producing diverse proteins, thus increasing species diversity. Investigating the molecular mechanisms underlying alternative splicing is not only profoundly significant in understanding gene expression processes in plants but also holds substantial medical relevance that cannot be overlooked.

### Alternative splicing in plants

Interestingly, alternative splicing is particularly abundant in plants [15, 16]. Notably, the pre-mRNA of numerous spliceosomal proteins, especially the serine/arginine rich (SR) proteins, undergo extensive alternative splicing. Recent studies have highlighted that alternative splicing in plants serves as a crucial post-transcriptional regulatory mechanism, modulating gene expression and ultimately influencing plant morphology and function. Alternative splicing impacts various essential plant processes, including photosynthesis, defense responses, and the regulation of flowering and fruiting quality [17].

Alternative splicing events in plants encompass a variety of mechanisms. Previous studies have identified seven distinct types that are widely observed [18–20] (Fig. 3). They include the most abundant exon skipping (ES) events observed in animals, wherein an exon is either included or excluded from the mature transcript, leading to the connection of two independent exons during the transition from the mRNA precursor to mature mRNA. Another type of alternative splicing is mutually exclusive exons (MEs), which involve a highly intricate process. This phenomenon entails two adjacent exons not being simultaneously present in the final mRNA; rather, one of them is excised. The third type is the retained intron event, wherein some or all of the introns that should have been excised are retained and can be observed in the final mature transcripts, thereby influencing subsequent physiological activities of plants. Notably, in *Chlamydomonas reinhardtii* the intron retention may enhance patchoulol production [21]. In current research, intron retention events in plants have been extensively investigated. Retained introns in mature transcripts serve two primary functions: increasing mRNA accumulation and influencing protein localization. However, intron retention is debatable compared with the common effects of alternative splicing, which can enhance protein diversity [22]. Alternative splicing can result in two mature transcripts of a gene differing either at the first exon or the last exon. These two types are termed alternative promoter (AP) and alternative terminator (AT). Additionally, two conventional types of alternative splicing exist. One is known as alternative acceptor site (AA), also referred to as 3′ alternative splicing, wherein the 5′-end splicing site remains the same in mature transcripts but the 3′-end splicing site varies, leading to elongation of the 3′-end exon in transcripts with alternative acceptor sites. Corresponding to AA is alternative donor site (AD), also known as 5′ alternative splicing, where the 3′-end splicing sites in mature transcripts are consistent but the 5′-end splicing sites differ, resulting in extension of the 5′-end exon in transcripts with alternative donor sites. However, with ongoing advancements in science and technology, researchers have identified an unconventional new type of alternative splicing, constituting the eighth kind, known as exitron splicing (EIS) [23, 24]. Studies have revealed the existence of a cryptic intron, termed an exitron, in EIS. These exitrons reside within exons’ internal regions and possess both protein-coding and splicing potential, exhibiting characteristics of both exons and introns. Research has confirmed the significant role of EIS in cancer prevention and treatment [23, 24].

Numerous large-scale studies have demonstrated that alternative splicing can occur across various tissues and developmental stages in plants, exhibiting dynamic changes that are dependent on the stage [25–28]. For instance, during the early stage of soybean development, a significant number of alternative splicing events occur, impacting the maturation process of the embryo [29]. Similarly, as *Arabidopsis* seeds mature, there is an observed increase in alternative splicing events [30], with their regulatory role being amplified to facilitate seed germination. Notably, mRNA splicing in plastids and mitochondria appears to be crucial for seed development and plant growth in *Arabidopsis* and maize [31–37]. Furthermore, it is noteworthy that alternative splicing is regulated by light signals, as revealed by RNA-seq analyses, thus influencing plant growth and development [38, 39]. Hence, the response of seedling development to light represents a significant regulatory process for alternative splicing. In addition, metabolic signals, particularly sugars, have been shown to be intricately linked to light-mediated alternative splicing regulation [40]. Furthermore, in *Arabidopsis* the coupling of nonsense-mediated decay (NMD) with alternative splicing events plays a crucial role in preserving the stability of the transcriptome [41, 42]. Additionally, in *Phaseolus vulgaris* alternative splicing produces two distinct subtypes of starch branching enzymes with differing properties, resulting in changes in their localization within starch granules [43]. These examples underscore the indispensable and decisive roles that alternative splicing events play in plants at both RNA and protein levels [44].

**Figure 4:**
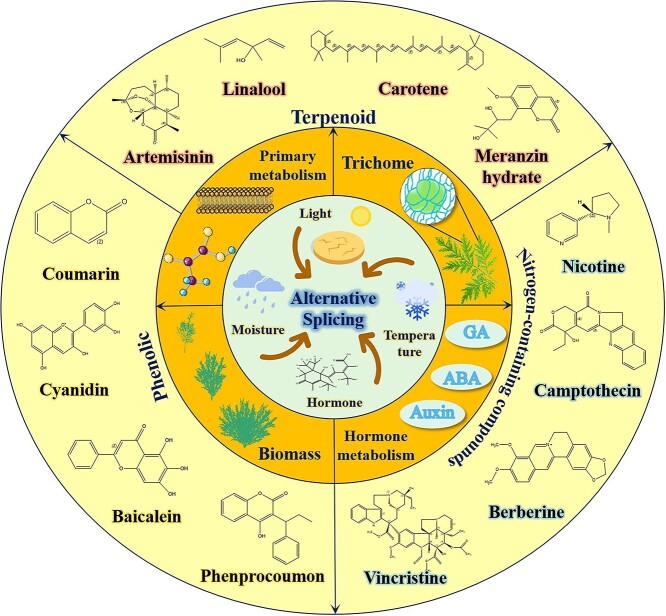
Exogenous signals induce the occurrence of alternative splicing events and play a significant role in plants. Signals such as light, moisture, temperature, and hormone levels trigger the formation of alternative splicing events in plants. Subsequent alternative splicing events further impact primary metabolic processes, biomass accumulation, hormone metabolism, and trichome density, ultimately influencing the accumulation of essential secondary metabolites in plants. Plants produce a diverse array of active ingredients that have been identified and isolated, organized according to their biosynthetic pathways. The terpenoid compound group includes artemisinin, linalool, carotene, meranzin hydrate, and others; the nitrogen-containing compound group comprises nicotine, camptothecin, berberine, vincristine, and others; the phenolic compound group encompasses coumarin, cyanidin, baicalein, phenprocoumon, and others.

Based on the aforementioned studies, alternative splicing events in plants are prevalent and contribute significantly to plant growth and development. In higher plants, alternative splicing is not only influenced by stressors like drought, light, and low temperatures [45–48], but also responds to varying hormone levels. This modulation affects primary metabolism, biomass, and downstream hormone metabolism, consequently impacting the accumulation of secondary metabolites such as terpenes, flavonoids, and nitrogen-containing compounds [10]. Thus, elucidating the relationship between alternative splicing and secondary metabolites is paramount (Fig. 4).

## Current understanding of regulation of alternative splicing in plant secondary metabolism

Alternative splicing is recognized as an essential regulatory mechanism for plant growth, development and adaptation to various biotic or abiotic stress conditions worldwide. However, research on the regulation and impact of alternative splicing on plant secondary metabolism lacks systematic investigation and summarization. The effects of alternative splicing on secondary metabolism will be elaborated in the following sections, categorized according to different types of secondary metabolites [10].

### Terpenoid metabolism regulation

Terpenes are isoprene polymers and their derivatives, with a skeleton based on C5 units. Mevalonate/mevalonic acid (MVA) serves as a crucial precursor compound in the terpene pathway. Research into the synthetic pathways of terpenoids reveals that they are synthesized through either the cytosolic mevalonate pathway or plastid dimethylerythritol phosphate pathway [10]. Terpenes can be classified based on the number of isoprene units they contain, resulting in categories such as monoterpenes, sesquiterpenes, diterpenes, and so forth. They are ubiquitous in nature and serve as primary constituents in various plant essences, resins, pigments, and more. Terpenes exhibit numerous physiological functions, including expectorant, antitussive, wind-repelling, sweating, insect-repelling, pain-relieving properties. For instance, artemisinin, a distinctive and vital component found in *A. annua*, is a terpene renowned for its potent antimalarial effects (Fig. 4).

Terpenoids confer distinct odors on plants due to their unique volatile characteristics, playing a crucial role in airborne signaling among organisms in nature and other biological interactions. In the tea plant (*Camellia sinensis*) the terpene synthase gene *CsLIS*/*NES* undergoes alternative splicing, yielding two distinct splice variants: *CsLIS*-*/NES-1* and *CsLIS*/*NES-2*. These variants exhibit differences in their expression patterns, including cellular localization and final expression products, attributed to the deletion of the N-terminal sequence in one of the transcripts and structural disparities. *CsLIS-NES-1* exhibits high expression levels in chloroplasts, primarily facilitating linalool biosynthesis. Conversely, *CsLIS/NES-2* is predominantly expressed in the cytoplasm and participates in nerolide biosynthesis. Therefore, the transcriptional regulation of these two distinct splice variants effectively governs the biosynthesis of sesquiterpene linalool and nerolide in the tea plant [49]. In *A. annua* it has been observed that there are numerous alternative splicing events in the upstream genes of the artemisinin biosynthesis pathway, such as farnesyl diphosphate synthase (FDS), 1-deoxy-d-xylulose-5-phosphate reductoisomerase (DXR) and 1-deoxy-d-xylulose-5-phosphate synthase (DXS), predominantly in the form of intron retention (IR). Additionally, premature termination codon (PTC) is detected within the retained introns of the IR gene, which is associated with the artemisinin biosynthesis pathway. These findings suggest that IR plays a role in regulating the expression of sesquiterpene metabolism [38], providing direct evidence that alternative splicing of pathway genes modulates sesquiterpene biosynthesis.

Alternative splicing can impact the biosynthesis of diterpenes. In *Oryza sativa*, transcription factors *OsWRKY62.1* and *OsWRKY76.1* have been found to suppress the biosynthesis of diterpene compounds such as momilactone A, phytocassane B, and phytocassane C. The truncated splice variants *OsWRKY62.2* and *OsWRKY76.2* have been observed to interact with each other, leading to a reduction in repressor activity in the biosynthesis of diterpenes. Consequently, alternative splicing of WRKY transcription factors can enhance plant defense responses by promoting the production of secondary metabolites [50]. Genes involved in the diterpene biosynthesis pathway undergo alternative splicing events, exemplified by class I terpene synthase (*IrKSL3*). This gene loses 18 nucleotides, resulting in the generation of the splicing variant *IrKSL3a*. *IrKSL3a* has the capacity to produce isopimaradiene and miltiradiene, whereas *IrKSL3* exclusively generates miltiradiene. [51].

Carotenoids are derived from isoprenoid molecules and belong to the terpene group [52]. High-tillering and dwarf 12 (*HTD12*) encodes a 15-*cis*-ζ-carotene isomerase (Z-ISO). *HTD12* can undergo alternative splicing, resulting in a splice variant, *htd12*, which lacks a 49-amino acid segment. This variant inhibits the biosynthesis of carotenoid in *O. sativa* [53]. Phytoene synthase (PSY) is the key enzyme responsible for producing carotene. PSY exhibits two alternative splice variants (ASVs). *ASV1*, characterized by a long 5′ UTR, produces carotene during the procession of plant development, whereas *ASV2*, featuring a short 5′ UTR, generates carotene rapidly upon induction in *Arabidopsis* [54]*.*

### Phenolic compound metabolism regulation

Phenolic compounds are hydroxy derivatives of aromatic hydrocarbons, predominantly synthesized as a result of plant metabolic activities. These compounds encompass a diverse array, including phenylpropanes, coumarins, flavonoids, and numerous others. Phenylpropanes can be synthesized via the shikimic acid pathway or malonic acid route. Research indicates that phenolic compounds in plants exist in two forms [55]: free phenolic compounds and bound phenolic compounds, which interact with macromolecular compounds such as proteins, cellulose, lignin, and carbohydrates. They exhibit distinctive physiological activities, including anti-oxidation, antibacterial, anticancer, antifungal and antiviral properties [56–58]. Moreover, they play pivotal regulatory roles in plant growth, development and signal transduction under stress conditions. Notably, phenolic acids, coumarins, and flavonoids confer significant resistance to plant pathogens [59] (Fig. 4).

It has been observed that the regulation of MYB in secondary metabolism in plants primarily centers on the synthesis of flavonoids and organic acids [60]. *SIAN2* encodes an R2R3-MYB transcription factor that plays a crucial role in regulating anthocyanin biosynthesis in tomatoes. It serves as a key gene in the anthocyanin biosynthesis pathway. The alternative splicing of *SIAN2* in wild-type tomatoes results in the complete loss of protein function, which ultimately determines the fundamental difference in anthocyanin expression between the two varieties. In wild-type tomatoes, *SIAN2* fails to form an MBW complex with basic helix–hoop–helix (bHLH) due to the absence of the R3 domain, leading to an early stop codon and the production of a non-functional mature transcript. However, *SIIAN2* in tomatoes is a functional splice variant involved in the formation of the anthocyanin biosynthesis complex, with significant interaction with the bHLH factor *SLAN1* [61]. MYB transcription factor *VvMYBA1* promotes the accumulation of anthocyanin in grapes. Alternative splicing of *VvMYBA1* generates a variant, *VvMYBA1-L*, which plays an opposite role in anthocyanin biosynthesis, thereby inhibiting flesh coloration in grapes and petal coloration in grapevines [62]. Similarly, another grape TF, *MYBA6*, can undergo splicing to produce *MYBA6.1*, which lacks an *MYC1* interaction domain and consequently cannot activate anthocyanin biosynthesis [63]. In sweet potato (*Ipomoea batatas* cv ‘Sinzami’), *IbMYB1* exhibits two splicing variants, namely *IbMYB1a* (249 amino acids) and *IbMYB1b* (104 amino acids). *IbMYB1a* has the ability to induce the expression of the structural genes in the anthocyanin biosynthetic pathway, resulting in the production of cyanidin in tobacco leaves. *IbMYB1b* retains the second intron, lacks a transcriptional activation domain, and may consequently form an inactive complex [64]. However, whether the *IbMYB1b* is non-functional or merely a non-functional pseudogene requires further investigation. This is because the intron retention may result in retention of the intron without encoding amino acids, and introns can enhance transcript expression and mRNA translation efficiency, thereby increasing mRNA stability [65]. In the second intron of *IbMYB1*, four NGATY core motifs were found, which play important roles in enhancing mRNA levels in a dose-dependent manner [66]. Therefore, we hypothesize that the second intron in *IbMYB1* may not encode amino acids. Hypothesizing that *IbMYB1b*, which contains the second intron and is full length, does not exclude the possibility that the full-length *IbMYB1b* has a stronger function than *IbMYB1*. However, it is important to note that not all the introns can enhance mRNA levels. Recent discoveries indicate that introns can play a negative role in gene expression. An alternative splicing event occurs in *cBrMYB2*, generating *gBrMYB2*, which lacks a large fragment in intron 1. Overexpression of *gBrMYB2* in *Arabidopsis*, with the shortened intron 1, results in a purple phenotype by promoting anthocyanin biosynthesis, while *cBrmyb2*, with a longer intron 1, produces a normal color in *Arabidopsis* [67, 68]*.*

Genes involved in the anthocyanin biosynthesis pathway typically undergo alternative splicing [68]. Dihydroflavonol-4-reductase (DFR) is a key enzyme in anthocyanin biosynthesis. Alternative splicing of the second intron of DFR may inhibit the accumulation of anthocyanin [69, 70].

In recent years, it has been reported that bHLH transcription factors can regulate the secondary metabolites of plants. For example, *CmbHLH2* in chrysanthemum produces two different structural transcripts due to the alternative splicing event, namely, full-field type *CmbHLH2* and truncated type *CmbHLH2*. The difference between the two cleavers is evident in the sequence structure, function and cellular distribution. The truncated form of *CmbHLH2* lacks a portion of the sequence, retaining only part of the interaction region with MYB, localizes in the cytoplasm, and is unable to interact with *CmMYB6*. In contrast, the full-length *CmbHLH2* can activate anthocyanin biosynthesis genes and induce pigment accumulation in transiently transfected tobacco leaves. Hence, distinct splice variants of *Chrysanthemum CmbHLH2* can modulate the deposition of flavonoid pigments in seeds and leaves by interacting with *CmMYB6* in chrysanthemum ray florets [71].

### Nitrogen-containing compound metabolism regulation

Nitrogen-containing compounds are widely distributed in nature. They are organic compounds with carbon–nitrogen bonds, synthesized primarily from amino acids. These compounds mainly include amines, alkaloids, and non-protein amino acids. The majority of nitrogen-containing compounds are primarily alkaloids, serving as intermediate products of nitrogen metabolism in plants. They exhibit diverse structures, significant pharmacological effects, and various physiological activities. Alkaloids like camptothecin, extracted from *Camptotheca acuminata*, demonstrate potent anticancer and antitumor properties. Additionally, vinblastine and vincristine, isolated from the plant *Vinca rosea* of the Oleaceae family, are effective in treating Hodgkin’s disease, choriocarcinoma and lymphosarcoma, exhibiting high efficacy and low side effects [72]. Furthermore, berberine and proberberine, isolated from *Berberis* and other plants, possess antibacterial and antifungal properties [73]. These findings suggest that alkaloids exhibit strong adaptability to both biotic and abiotic stresses (Fig. 4).

The pathogenesis-related 3b protein gene (*PR3b*) transcript in burley tobacco, a gene associated with disease, undergoes premature termination codon (PTC) introduction via alternative splicing, thereby altering the length of the amino acid sequence and reducing the transcript’s abundance. This also implies that the specific enzyme activity of tobacco *PR3b*, acting as a plant chitinase, is significantly diminished, leading to a substantial reduction in nicotine content within the leaves [74]. Another example involves strictosidine β-d-glucosidase (SGD), which participates in the synthesis of cytotoxic monoterpene indole alkaloids (MIAs) in *C. roseus*. The alternative splicing of the final exon generates two different subtypes, thereby influencing enzyme activity. Consequently, the author contends that the alternative splicing of SGD represents a dependable mechanism for regulating the biosynthesis of monoterpene indole alkaloids [75]. In summary, the alternative splicing of transcription factors impacts the synthesis of nitrogen-containing compounds, such as alkaloids, among the secondary metabolites of plants.

To sum up, numerous transcription factors, including MYB and bHLH transcription factors, undergo extensive and diverse alternative splicing events, as confirmed by experiments, and are widely acknowledged. Simultaneously, transcription factors play a crucial role in regulating plant secondary metabolites. Therefore, it is reasonable to infer that the alternative splicing of transcription factors might impact the biosynthesis and accumulation of plant secondary metabolites. Even if it cannot directly regulate secondary metabolism, for example, JASMONATE ZIM-domain (JAZ) protein, through alternative splicing, can indirectly influence secondary metabolites by interaction with MYB transcription factors. However, currently, there is a scarcity of research on the direct impact of alternative splicing of transcription factors on secondary metabolism. Therefore, in the future, more emphasis can be placed on investigating the influence of alternative splicing of transcription factors on secondary metabolism, which presents a new avenue for future research.

## Alternative splicing-mediated phytohormone regulation of secondary metabolites

Plant hormones are organic substances produced by a plant’s metabolism; they move from the producing part to the acting part and have a significant physiological effect even at very low concentrations. These hormones are also known as natural plant hormones or endogenous plant hormones. Plant hormones can be categorized into auxin, gibberellin (GA), cytokinin (CTK), abscisic acid (ABA), jasmonic acid (JA), salicylic acid (SA), ethylene (ETH), and brassinosteroids (BR), among which cytokinins play a crucial role. Phytohormones serve as important regulators in modulating the content of secondary metabolites [76, 77]. According to numerous reports, selective splicing events occur in enzymes and genes involved in plant hormone signaling, thereby regulating the synthesis and accumulation of secondary metabolites (Fig. 4).

### Alternative splicing occurs in the jasmonic acid biosynthetic pathway and signaling transduction pathway

**Figure 5:**
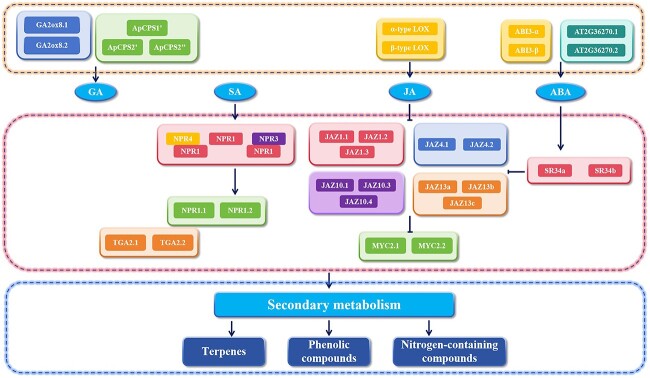
Model diagram illustrating the influence of alternative splicing of key pathway genes and enzymes in common hormone pathways on secondary metabolites.

JA is an endogenous growth regulator in higher plants, playing a crucial role in plant defense against harsh environmental conditions and challenges from biotic and abiotic factors. It has been acknowledged as one of the primary plant hormones regulating stress responses by activating defense mechanisms and triggering the production of specialized metabolites (Fig. 5).

In the JA biosynthetic pathway, alternative splicing commonly occurs. The key enzyme gene, lipoxygenase (*LOX*), plays crucial roles in JA biosynthesis. In *C. sinensis*, six *CsLOX* genes undergo alternative splicing, resulting in the production of two variants for each *CsLOX*. Each *CsLOX* gene has two types: α-type and β-type. The α-type and β-type *CsLOX*s exhibit distinct expression patterns and may compete or compensate in their regulation of each other, thereby influencing the content of JA and altering the levels of secondary metabolites [78].

The alternative splicing of JAZ affects the accumulation of secondary metabolites. In the JA signaling transduction pathway, JAZ, a transcriptional inhibitor, is a key regulatory factor in the plant’s response to JA signals [79]. JAZ protein consists of two conserved domains: the ZIM domain and Jas domain at the C-terminal [79]. Using JAZ10 as an example, the various variants resulting from its alternative splicing are closely associated with differences in the Jas domain. This process primarily yields three transcripts: full-length JAZ10.1, partially truncated JAZ10.3, and JAZ10.4, which lacks the Jas domain entirely [80]. JAZ10.4, an alternative splice variant of JAZ10 lacking the Jas motif, is capable of interacting with MYC2, MYC3, and MYC4. Upon induction by JA, the protein levels of JAZ10.4 increase, leading to the attenuation of signaling; this variant may inhibit the biosynthesis of downstream secondary metabolites regulated by MYC2, MYC3, and MYC4 [81]. Therefore, JAZ splice variants regulate secondary metabolites through negative feedback regulation of JA signaling.

The primary function of the jas motif is to interact with the transcription factor MYC2, inhibiting JA signal transduction and participating in the degradation of JAZ. It serves as a key domain essential for plants to respond to JA signals. Hence, the deletion of the Jas motif in JAZ10.4 results in a phenotype characterized by JA insensitivity, male root sterility, and reduced root elongation in response to JA. Studies have indicated that JAZ negatively regulates anthocyanin biosynthesis in plants by interacting with MYB and bHLH subunits of the MBW complex [82].

The alternative variants of JAZs primarily interact with *MYC2* to regulate secondary metabolites. *MYC2* serves as the key gene regulating the biosynthesis of secondary metabolites in plants. JAZ splice variants typically interact with *MYC2* to suppress or dampen the biosynthesis of secondary metabolites. In *C. sinensis*, *CsJAZ1* has three splice variants, *CsJAZ1-1* (full-length), *CsJAZ1-2* (lacking 30 coding sequences), and *CsJAZ1-3* (lacking the coding region for the Jas domain). Under JA induction, *CsJAZ1-3* interacts with *CsJAZ1-1* and *CsJAZ1-2* to form the *CsJAZ1-3–CsJAZ1-1–CsMYC2* and *CsJAZ1-3–CsJAZ1-2–CsMYC2* complexes, respectively, thereby repressing the gene expression involved in the biosynthesis of flavan-3-ol [83]. In addition, the splice variant ΔPYJAZ6 of JAZ6 competes with MED25 for binding to MYC2 [79], thereby affecting the accumulation of secondary metabolites.

The alternative variants of JAZs can regulate secondary metabolites through a feedback loop. The JAZ splice variant may control JA signaling by negative feedback regulation, thereby modulating the accumulation of secondary metabolites. In rice, *OsJAZ13* has three different splice variants: *OsJAZ13a*, *OsJAZ13b*, and *OsJAZ13c* [84]. These different splice variants regulate the JA response through a feedback regulation mechanism. When JA signaling is induced, JAZ splice variants containing an N-terminal cryptic MYC-interaction domain (CMID) are produced, which cannot recruit SCF^COI1^, leading to accumulation of CMID-containing JAZ splice variants [85].

### Alternative splicing occurs in the abscisic acid pathway

ABA also promotes the biosynthesis of secondary metabolites [86]. The alternative splicing of genes in the ABA signaling pathway can indirectly affect the secondary metabolism of plants by influencing the hormone levels. Recently, numerous reports have indicated that alternative splicing occurs in the ABA signaling pathway. The splicing factor ski-interacting protein (SKIP) can bind to the pre-mRNA of ABA signaling pathway genes, such as *ABSCISIC ACID INSENSITIVE 1* (*ABI1*), *ABSCISIC ACID INSENSITIVE 5* (*ABI5*), *pyrabactin resistance-like 7* (*PYL7*), and *pyrabactin resistance-like 8* (*PYL8*), to modulate their splicing, thereby affecting the accumulation of ABA content [87]. In *Arabidopsis*, *ABI3* and *ABI5* have two splice variants each: *ABI3-α*, *ABI3-*β, and *AT2G36270.1*, *AT2G36270.2* [88, 89], respectively. The various splicing variants in the ABA signaling pathway alter ABA content, thereby regulating the biosynthesis of secondary metabolites such as artemisinin and anthocyanin [86, 90] (Fig. 5).

### Alternative splicing occurs in other phytohormone pathways

SA can induce the biosynthesis of secondary metabolites in plants, such as artemisinin and tanshonetone [76, 77]. Alternative splicing commonly occurs in the SA signaling pathway. In the biosynthetic pathway of SA, isochorismate synthase (ICS) is an important enzyme for SA synthesis. In *Populus*, ICS genes undergo alternative splicing, altering the accumulation of phenylpropanoids [91] and affecting the content of secondary metabolites in downstream pathways (Fig. 5).

GA may influence the biosynthesis of artemisinin in *A. annua*, as well as affecting the content of flavone [92, 93]. Genes in the GA biosynthesis pathway can also undergo alternative splicing. Gibberellin 2-β-dioxygenase 8 (GA2ox8) is a key enzyme gene involved in the biosynthesis of GA and contains two splice variants, GA2ox8.1 and GA2ox8.2. GA2ox8.2 has an intron retention between the second and third exons, deactivating the bioactivity of GAs compared with GA2ox8.1 [94]. In *Salvia miltiorrhiza*, SmGA20ox3, SmGA2ox3, and SmGA2ox11 exhibit different splice variants that regulate the biosynthesis of tanshinone [95]. Additionally, genes encoding diterpene synthases (ApCPS1 and ApCPS2) undergo splicing to produce variants ApCPS1′, ApCPS2′, and ApCPS2″, respectively. While ApCPS1 is involved in the biosynthesis of GA, ApCPS2 may influence the biosynthesis of geranylgeranyl diphosphate (GGPP). Consequently, ApCPS1, ApCPS2 and their splicing variants collectively regulate the content of diterpene in *Andrographis paniculata* [96]*.*

## Stress-mediated alternative splicing regulates secondary metabolism

Common environmental stresses include salt stress, drought stress, temperature change, light, etc. These factors not only impact plant growth but also influence the synthesis of secondary metabolites [97] (Fig. 4).

Indeed, certain spliceosome components have been observed to react to abiotic stresses. In recent years, an increasing body of research has highlighted the pivotal role of plant alternative splicing in regulating plant growth and responses to external stresses [98, 99].

It has been reported that alternative splicing in *Arabidopsis* can impact the transcription factor involved in stress response, namely Independent Domain 14 (*IDD14*), subsequently influencing starch expression levels. Previous studies have demonstrated alternative splicing events in *IDD14*, with the IDD14α subtype encoding transcription factors. These factors bind to the promoter of Qua-Quine Starch (*QQS*), activating downstream *QQS* genes and regulating starch accumulation in plants through interaction. However, *IDD14β* lacks a DNA-binding domain, rendering it unable to perform this function. Instead, it shares similarities with *IDD14α* and forms dimers, which impede normal starch biosynthesis [100]. In recent years, researchers have discovered that SR45a directly binds to the first intron of *IDD14* pre-mRNA in high-salt environments, leading to increased alternative splicing events. This increase in the *IDD14β*/*IDD14α* ratio results in reduced transcription of downstream *QQS* genes and a corresponding decrease in starch synthesis [101]. A similar scenario occurs in *Arabidopsis* under cold stress, where the *IDD14β* subtype decreases *QQS* transcription, consequently altering starch accumulation [100]. Recent studies have confirmed the specific role of *SmEb*, a core spliceosome mediating alternative splicing in *Arabidopsis thaliana* [102]*. SmEb* acts as a positive regulator in response to salt stress, and plant salt tolerance is diminished in *smeb* mutants. Moreover, *SmEb* is implicated in the regulation of numerous alternative splicing events of pre-mRNA, thereby modulating salt tolerance in plants through this pathway. In *A. thaliana*, *RCD1* functions as a cell death regulator that protects plant cells by mitigating oxidative damage induced by environmental stress [103]. *RCD1* interacts with the splicing protein DREB2 to modulate plant development, hormone signaling, and stress responses [102, 104]. Under high salt conditions, the upregulation of both isoforms of *DREB2* enhances plant salt tolerance. These findings imply that proper splicing of RCD1 contributes to elevated salt tolerance and the preservation of normal physiological functions in plants under salt stress. However, in *smeb* mutants where the core factor *SmEb* is deleted, there is a heightened likelihood of erroneous splicing, leading to the accumulation of IR and alternative splicing events such as exon skipping. This alteration results in a shift in the ratio of the two splice isoforms of *RCD1*, with an increase in *RCD1.2* content and a decrease in *RCD1.1*. Therefore, the researchers concluded that *SmEb*, as a spliceosome component, holds significant importance in preserving the appropriate alternative splicing of pre-mRNA crucial for plant responses to salt stress [102]. Additionally, the accuracy of *RCD1* alternative splicing is paramount for conferring resistance to salt stress, underscoring the intricate connection between alternative splicing and plant adaptation to environmental stressors.

## Alternative splicing regulates secondary metabolism by affecting plant biomass accumulation

Plant biomass is a pivotal trait in productivity, and its allocation pattern is a crucial topic in plant ecology and evolutionary studies. Alternative splicing events have been implicated in biomass regulation in plants. The sugarcane R2R3-MYB gene (*ScMYBAS1*) exhibits four alternative splicing subtypes, namely *ScMYAS1-2*, *ScMYBAS1-3*, *ScMYBAS1-4*, and *ScMYBAS1-5*, each displaying distinct physiological activities. The results indicate that overexpression of *ScMYBAS1-3* in transgenic rice lines led to an increase in biomass (total dry weight) and facilitated the growth and development of sugarcane under both normal watering and drought conditions. These findings suggest a close association between *ScMYBAS1-3* and plant drought tolerance as well as biomass enhancement. It is widely recognized that alternative splicing events frequently occur in MYB transcription factors [105]. Therefore, it is reasonable to infer that alternative splicing of MYB transcription factors could impact the secondary metabolism of plants, consequently influencing plant biomass accumulation. Recent technological advancements have indeed supported this inference through experimental validation. Overall, alternative splicing events are prevalent across organisms and exert an influence on the accumulation of crucial substances in plants by modulating biosynthesis pathways (Fig. 4).

## Primary metabolism mediated by alternative splicing affects the accumulation of secondary metabolites

Primary metabolism encompasses carbohydrates, amino acids, fatty acids, nucleic acids, and their corresponding polymers synthesized through primary metabolic pathways, which are prevalent in most organisms in nature and share similarities in their metabolic pathways and accumulated primary metabolites [106]. However, various organisms inhabit different environments and ecological niches, underscoring the significance of secondary metabolic pathways. While conservative primary metabolism remains essential, diversified specialized metabolism furnishes the metabolic foundation for plants’ adaptation to their environment and normal growth. This elucidates the intricate relationship between primary and secondary metabolism [107]. Studies indicate that the occurrence of alternative splicing events in genes involved in primary metabolic pathways can either increase or decrease. This regulation modulates the synthesis and accumulation of primary metabolites, consequently impacting the expression of plant secondary metabolites (Fig. 4).

Starch, a primary metabolic product, serves as the principal product and energy storage material resulting from plant photosynthesis. It also constitutes the main energy source for humans and animals, playing an indispensable role [108]. Fruit ripening involves a series of intricate biological processes, comprising plant hormone metabolism and signaling, cell wall degradation, formation of flavor and aroma compounds, and pigment biosynthesis and degradation. A comprehensive comprehension of the mechanisms governing fruit ripening is crucial for devising strategies to enhance fruit quality and prolong shelf life. Jiang *et al*. discovered, for the first time, that R1-type MYB-type transcription factors can undergo alternative splicing events, consequently influencing the accumulation of the primary metabolite, starch, thereby modulating fruit ripening [109]. In bananas (*Musa acuminata*), the transcription factor *MaMYB16* undergoes selective splicing to generate two isoforms, *MaMYB16L* and *MaMYB16S*. The full-length *MaMYB16L* isoform is capable of binding to the promoter regions of genes associated with starch degradation, thereby impeding starch breakdown. Additionally, *MaMYB16L* hinders the binding of *MsDREB2*, a positive regulator of fruit ripening. [109]. Hence, throughout banana ripening, the expression level of *MaMYB16L* is decreased, while *MaMYB16S* exhibits an opposite trend, thereby facilitating the degradation of primary metabolite starch and accelerating the softening rate of banana fruit [109].

Furthermore, lipids are crucial components of animal and plant cell membranes, with their metabolic processes holding significant importance. Additionally, vegetable oil serves primarily as a liquid transport fuel with renewable properties, boasting considerable commercial value [110]. Phosphatidylic acid, an intermediate involved in lipid storage and membrane lipid synthesis [111], can be catalyzed by diacylglycerol kinase (DGK) [112]. It has been discovered that two distinct DGK isoforms can be generated in tomato (*Solanum lycopersicum*), both exhibiting catalytic activity of DGK. The full-length isoform, LeCBDGK, features a calmodulin binding domain at the C-terminal, facilitating binding to calcium ions [112]. The truncated isoform, LeDGK1, lacks this domain and is insensitive to calcium ions [112]. Therefore, tomato produces two DGK isomers with varying sensitivity to calcium ions through alternative splicing, thereby offering flexibility in response to calcium ions during physiological processes [112].

As we understand, plant primary metabolism, driven by processes like photosynthesis and the citric acid cycle, supplies crucial energy and small molecular compounds for secondary metabolism. Consequently, the alternative splicing of pivotal genes in the primary metabolic synthesis pathways in plants not only impacts the accumulation of primary metabolites but also indirectly increases or decreases the expression of secondary metabolites.

## Alternative splicing regulates secondary metabolism by affecting the density of plant trichomes

Trichomes are vital tissues for depositing secondary metabolites, serving as a defense mechanism in plants against insects and pathogenic microorganisms through physical or chemical means [113]. Alternative splicing events in plants can influence the density of trichomes, which serve as sites for the synthesis and accumulation of crucial secondary metabolites. For instance, artemisinin, a potent compound, is synthesized and stored in the secretory glandular hairs of *A. annua* [114]. Therefore, alternative splicing can impact the abundance of secondary metabolites.

The Nck-associated protein 1 (*NAP1*) gene, a positive regulator of trichome development, undergoes splicing to produce two short variants (AlNAP1-AS1 and AlNAP1-AS2) lacking multiple exons in *Actinidia latifolia*. However, neither AlNAP1-AS1 nor AlNAP1-AS2 can rescue the mutant phenotype of *nap1* mutant in *Arabidopsis* [115]. This suggests the existence of a feedback mechanism regulating trichome density through alternative splicing. The *BrAN* gene exhibits two splicing variants, *lhd1* and *lhd2*, both of which influence trichome branching in Chinese cabbage by causing abnormal cortical microtubule arrangement [116]. The *lhd1* variant is characterized by intron retention, suggesting the significance of the intron in trichome development. In *Arabidopsis* it has been observed that the second intron enhances the expression level of *GL3* [117]. The *MYB82* gene comprises two introns. Interestingly, while the coding sequence of *MYB82* alone fails to rescue the *myb82* mutant phenotype, *MYB82* containing both introns can successfully rescue it. However, *MYB82* with either of the two introns individually cannot fully rescue the *myb82* mutant [118]. The result is similar to that of a previous study indicating that an intron alone can fully restore the mutant phenotype [119]. This is because introns contain key *cis*-elements regulated by other factors for precise temporal and spatial expression.

The class IV homeodomain-leucine zipper (HD-Zip IV) protein may be involved in trichome development and typically undergoes alternative splicing [120]. In cucumber, the HDZIP transcription factors *MICT*, *TBH*, and *CsGL1* are allelic with alternative splicing, resulting in different phenotypes of trichomes under microscopic observation [121]. The negative regulator of trichome development has been found to undergo alternative splicing evens. The CAPRICE (CPC)-like MYB4 (*CPL4*), also known as *TCL2*, exhibits two splicing variants, *CPL4-α* and *CPL4-β*. These three splicing variants collectively regulate trichome development in *Arabidopsis* [122]*.* Splicing regulators can also influence the development. SmD3, an snRNP, plays crucial roles in the splicing of primary transcripts. *SmD3* has two splicing variants, *SmD3-a* and *SmD3-b*, both of them regulating trichome development [123].

## Conclusions and future directions

An increasing body of research indicates the significant role of alternative splicing in various aspects of plant life processes. These splicing events are not only crucial at the RNA and protein levels but also serve as indispensable regulatory mechanisms for the growth, development, and adaptation of all plant species to diverse environmental conditions. In nature, plants respond to environmental stress and fluctuations in hormone levels, often exhibiting a plethora of alternative splicing events in genes involved in signal transduction and synthesis pathways. In recent years there has been a growing recognition of the pivotal role of alternative splicing in plants, particularly in the regulation of growth and response to external stress. Alternative splicing is intricately linked to plant responses to environmental stress. Moreover, alternative splicing events regulate physiological and biochemical processes such as the synthesis and accumulation of crucial primary metabolites, biomass accumulation, hormone metabolism, and trichome density in plants, thereby influencing the expression levels of secondary metabolites. Overall, alternative splicing processes are ubiquitous in plants and play a significant role in secondary metabolism. Investigating alternative splicing events in plants can enhance our understanding of and precision in controlling secondary metabolic processes and the synthesis of secondary metabolites, thereby furnishing a reliable foundation for guiding future production endeavors.

However, current research on alternative splicing still faces limitations due to the lack of timely and continuous updates of public databases, resulting in a significant amount of potentially valuable data being classified as junk. Additionally, delays in the uploading of new transcripts by the research community can hinder the discovery of alternative splicing events. Theoretically, the entire transcriptome harbors an infinite number of splicing possibilities, with infinite potential conditions influencing alternative splicing and promoting the emergence of new isoforms. A more direct approach involves manually assembling transcripts from specific genes. Manual mapping, while a low-throughput method that is time-consuming and inefficient, offers the highest probability of identifying all transcripts from a single gene [124]. Hence, the primary objective of future alternative splicing research will be to efficiently and precisely identify specific events within the transcriptome. Simultaneously, there is considerable scientific interest in functionally characterizing all alternative splicing isoforms of each gene and discerning their roles in regulating various metabolic processes, thus elucidating the underlying mechanisms of alternative splicing [44]. In summary, investigating the role of alternative splicing in regulating plant metabolism will furnish crucial foundational knowledge and offer novel approaches and insights for enhancing plant performance, yield, and utility through bioengineering.

## Data Availability

Data availability is not applicable to this article as no new data were created or analyzed in this study.
